# Key lessons from integrated care in Europe

**DOI:** 10.5334/ijic.830

**Published:** 2012-01-30

**Authors:** Jon Glasby

**Affiliations:** Health Services Management Centre, University of Birmingham, UK

In English health and social care there sometimes seems to be a curious two-fold response when a difficult policy issue arises. First is an assumption that the answer to the problems we’re facing must exist somewhere—we just haven’t it yet. This then leads to a rapid trawl of international models and experiences to see if we can find and import a ready-made solution. Secondly, when England looks at international systems it seems to focus more on US examples of good practice (and to a lesser extent experience from Canada, New Zealand and Australia). Typically, it feels as if there is less learning from European approaches, perhaps reflecting a slightly ambivalent relationship with the European Union more generally.

These two issues have always struck me in my work as a UK policy analyst and commentator. However, as a partner in a large EU-funded study into long-term care for older people, I have been reminded of both tendencies once again. The project—known as INTERLINKS—is described in more detail by Kai Leichsenring in this journal http://www.ijic.org/. However working on a 15-partner, 13-country study such as this has taught me three main lessons:

- Although different countries seem to have very different traditions, structures and approaches (at face value), the underlying problem of non-joined up health and social care seems remarkably consistent across countries. To the policy-makers who are looking for instant answers, the unfortunate truth is that everyone seems to be struggling with the same issues. Although this can seem a little depressing, it may actually be reassuring for busy practitioners and managers. Perhaps there are no easy answers because the problems we face are genuinely difficult—and if they were simple we’d have resolved them by now.
- Many of the approaches that different countries are developing also seem remarkably similar—whether it be case management, single assessment, integrated teams, new professional roles, an emphasis on more efficient and timely hospital discharge, and so on. For all that policy-makers may look for ‘new’ ideas, perhaps what’s possible to do and think at any one time is so constrained politically, socially and economically that even apparently different systems end up exploring the same basic approaches and ideas.
- Despite all this (and indeed perhaps because of it), there is much that England can learn from its European partners. Thus, English policy-makers exploring financial incentives to improve hospital discharge can look to Sweden and Denmark, while those introducing personal budgets can learn from the experience in the Netherlands and Germany (to take but two examples). While countries like the US may have good practice to share, we miss a tremendous amount of learning if we look only (or even primarily) to America and neglect the expertise that exists across Europe.

In 2012, the English government has published a review by the National Health Service Future Forum [1] calling for more integrated care. If this is to become a reality, then we need to realise that:

- This is the goal for a range of different countries with a range of different histories and contexts.
- No one yet seems to have cracked it—and the search for a definitive solution continues.
- The ideas we’re debating in England have many similarities with approaches and experiences from other European countries.
- Projects like INTERLINKS which help different countries and partners to learn from each other are unlikely to produce a ‘magic answer’—but there remains much to learn from each other nonetheless.

For further details of the INTERLINKS project, see the article by Kai Leichsenring Integrated Care for older people in Europe–latest trends and perceptions on the IJIC website http://www.ijic.org/ or visit the INTERLINKS website via: http://interlinks.euro.centre.org/
